# Determination of the optimal injection parameters for the SPME Arrow analysis of VOCs

**DOI:** 10.1007/s00216-026-06757-y

**Published:** 2026-08-31

**Authors:** Nenad Stojanović, Maik A. Jochmann, Torsten C. Schmidt

**Affiliations:** 1 Instrumental Analytical Chemistry, University of Duisburg-Essen, Universitätsstrasse 5, 45141 Essen, Germany; 2 Centre for Water and Environmental Research, University of Duisburg-Essen, Universitätsstrasse 5, 45141 Essen, Germany; 3 IWW Water Centre, Moritzstrasse 26, 45476 Mülheim an der Ruhr, Germany

**Keywords:** SPME Arrow, SPME-GC-MS, Injection, Thermal desorption, Liner geometry, Volatile organic compounds

## Abstract

The injection step in SPME Arrow-GC-MS analysis is critical for sensitivity, repeatability, and peak shape, but is often less systematically optimized than extraction. In this study, the injection efficiency of SPME Arrow fibers was investigated using 1-methylnaphthalene as a model volatile organic compound. Real-time desorption-and-transfer profiles were recorded to evaluate the effects of liner geometry, carrier-gas flow rate, and column insertion depth for two coatings, PDMS/CWR and PDMS/DVB. Here, desorption denotes analyte release from the coating, while transfer denotes the subsequent transport of the released analyte through the injector and into the column or detector connection. Total peak area was used as a measure of transferred analyte amount, while the half-mass point described transfer kinetics. The results show that SPME Arrow injection is governed by a combined desorption-and-transfer process rather than by thermal desorption alone. For PDMS/CWR, a narrow 1.2 mm i.d. liner markedly improved injection efficiency compared with a conventional 2.0 mm i.d. liner, providing relative desorption-and-transfer response close to 100% over a broad range of carrier-gas flow rates. The narrow liner also reduced half-mass points, indicating faster transfer. For PDMS/DVB, however, the highest total peak areas were obtained with the wider liner at elevated carrier-gas flow rates, although the narrow liner produced faster transfer. These results demonstrate that optimal injection conditions are strongly coating-dependent and cannot be predicted solely from liner diameter or carrier-gas velocity. The study highlights the need to optimize SPME Arrow injection parameters separately for each coating and analytical objective. Both total transferred amount and transfer kinetics should be considered, since the fastest transfer condition does not necessarily provide the highest analyte recovery.

**Graphical abstract:**

**Figure:**
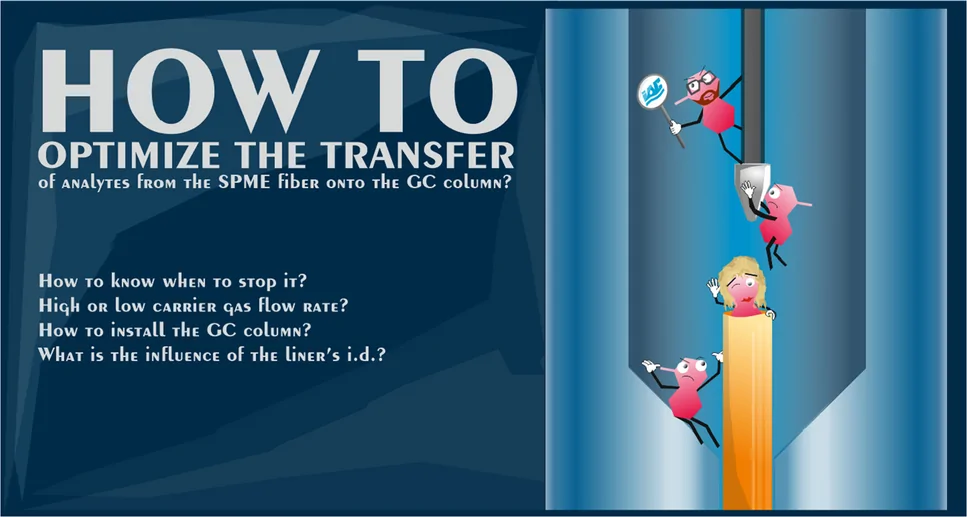

**Supplementary Information:**

The online version contains supplementary material available at https://doi.org/10.1007/s00216-026-06757-y.

## Introduction

Solid-phase microextraction (SPME) is a solvent-free sample preparation technique that integrates sampling, cleanup, enrichment, and, where required, derivatization into a single procedure. Since its introduction in the early 1990s, SPME has become widely established, particularly in combination with gas chromatography (GC), and has been applied in environmental, food, forensic, biomedical, and industrial analysis [1–6]. Its advantages include minimized sample handling, high enrichment capability, and suitability for automated workflows. These properties make SPME especially attractive for trace-level analysis of volatile and semi-volatile organic compounds in aqueous matrices [3, 7, 8].

Despite these advantages, conventional SPME fibers have limitations. Their mechanical fragility can reduce lifetime, especially in automated systems or complex matrices, and their comparatively small extraction-phase volume limits sorption capacity and sensitivity [9]. The SPME Arrow was introduced to overcome these drawbacks. Owing to its larger outer diameter, protected sorptive phase, and increased coating volume, it offers improved mechanical robustness and higher extraction capacity. Several studies have demonstrated enhanced sensitivity and robustness compared with conventional SPME fibers [10–14].

However, the larger dimensions of the SPME Arrow also alter the injection conditions in the GC inlet [12]. While SPME method development often focuses on extraction parameters such as coating chemistry, extraction time, temperature, agitation, ionic strength, sample volume, and phase ratio, analytical performance is also strongly affected by the injection step [9]. After enrichment, analytes must be efficiently released from the coating and transferred through the injector into the analytical column. Inefficient injection may therefore compromise sensitivity, peak shape, repeatability, and quantitative accuracy, even if extraction itself is efficient [15].

In this context, the term *desorption efficiency* is incomplete. For quantitative SPME-GC analysis, the relevant process includes both thermal release from the coating and transfer of the desorbed analytes through the inlet into the column. In this study, the relative desorption-and-transfer response was used as an operational comparative parameter based on the integrated peak area obtained after SPME Arrow injection. It reflects the combined effects of analyte release from the SPME Arrow coating and subsequent transfer through the injector liner into the transfer capillary. For each SPME Arrow coating, the injection condition giving the highest mean integrated peak area was defined as 100%, and all other conditions were expressed relative to this coating-specific reference. This distinction is important because complete release from the coating does not necessarily guarantee quantitative transfer to the analytical column. Desorbed analytes may be affected by dilution in the injector volume, insufficient carrier-gas velocity, adsorption on active surfaces, unfavorable column positioning, or losses through the split outlet.

Several instrumental parameters can influence this process. For conventional SPME fibers, narrow-bore liners are commonly recommended because they reduce free volume around the fiber, increase linear carrier-gas velocity, improve heat transfer, and promote rapid analyte transport toward the column inlet. These principles cannot be transferred directly to SPME Arrow without considering the annular gap between the Arrow surface and the liner wall. Owing to the larger outer diameter of the Arrow, the free cross-sectional area available for carrier-gas flow differs substantially from that in conventional SPME configurations, even when similar liner diameters are used. Consequently, the local carrier-gas velocity, residence time of desorbed analytes, and efficiency of transport toward the column inlet may differ markedly. Previous studies have investigated SPME desorption by connecting the injector directly to the mass spectrometer through a short deactivated capillary and recording desorption profiles in real time [16, 17]. Such approaches provide information on the rate and completeness of analyte release and transfer. Most available data, however, refers to classical SPME fibers. SPME Arrow may behave differently because of its greater coating volume, larger surface area, and altered inlet geometry.

The aim of this study was therefore to systematically investigate the injection efficiency of SPME Arrow fibers in a split/splitless GC injector. The effects of liner geometry, carrier-gas flow rate, and column insertion depth were evaluated using real-time desorption-and-transfer profiles, total peak areas, and half-mass points. By distinguishing between thermal desorption and complete analyte transfer, this work provides a more detailed understanding of the parameters controlling SPME Arrow injection and supports the development of robust SPME-GC-MS methods for volatile organic compounds.

## Materials and methods

### Fibers and chemicals

SPME Arrow fibers coated with polydimethylsiloxane/carbon wide range (PDMS/CWR, 120 μm × 20 mm) and polydimethylsiloxane/divinylbenzene (PDMS/DVB, 120 μm × 20 mm) were purchased from BGB Analytik GmbH, Rheinfelden, Germany (For dimensions of the SPME Arrow fibers see Table S3). Before use, fibers were conditioned for 60 min at 250 °C under helium. Before each measurement, they were reconditioned under the same conditions for 10 min. The SPME Arrow had an overall external diameter of 1.1 mm. The coating dimensions were 120 μm × 20 mm for both PDMS/CWR and PDMS/DVB with a sorptive phase volume of 3.8 µL.

The SPME Arrow was inserted 70 mm into the injector, corresponding to the maximum mechanically accessible insertion depth of the autosampler/injector configuration. This insertion depth was kept constant throughout all experiments. Both investigated coatings consist of a PDMS matrix containing adsorptive particles, but they differ in particle chemistry, morphology, and expected analyte-phase interactions. PDMS/CWR contains carbon-based sorptive material embedded in PDMS, whereas PDMS/DVB contains divinylbenzene particles within the PDMS matrix. Both coatings are suitable for volatile and semi-volatile organic compounds, but coating-specific differences in adsorption strength, diffusion pathways, and thermal release behavior may affect desorption-and-transfer kinetics. Therefore, the response to liner geometry, carrier-gas flow, and column insertion depth was expected to depend on the individual coating.

Ultrapure water was obtained from an ELGA Purelab Ultra system (Veolia Water Technologies, Ratingen, Germany). Standard solutions were prepared by adding 1 µL of 1-methylnaphthalene directly to a 1-L Schott bottle filled completely with ultrapure water, leaving no headspace to minimize partitioning into the gas phase.

Standard solutions were prepared by adding 1 µL of 1-methylnaphthalene directly to a 1-L Schott bottle filled completely with ultrapure water, leaving no headspace to minimize partitioning into the gas phase. Direct preparation from the neat compound was chosen to avoid organic co-solvents, which may influence analyte activity, SPME coating uptake, and surface interactions during extraction. Complete dissolution and equilibration were promoted by mixing the solution on an overhead shaker for 24 h at 13 rpm. The preparation and equilibration approach follows the procedure previously evaluated by Jochmann et al. [18]. The aqueous 1-methylnaphthalene concentration should be regarded as nominal because dosing 1 µL of the neat compound introduces additional uncertainty in the absolute analyte concentration. However, since the present study focused on relative desorption-and-transfer responses rather than absolute quantification, all injection conditions were compared using identically prepared aqueous standards and normalized peak areas.

1-Methylnaphthalene was selected because its physicochemical properties are representative of a volatile aromatic compound, while its comparatively high boiling point makes it a suitable test compound for evaluating desorption and transfer limitations. It therefore represents a suitable test compound for evaluating thermal desorption and analyte transfer during SPME Arrow injection.

### GC-MS measurements of desorption-and-transfer profiles

**Fig. 1:**
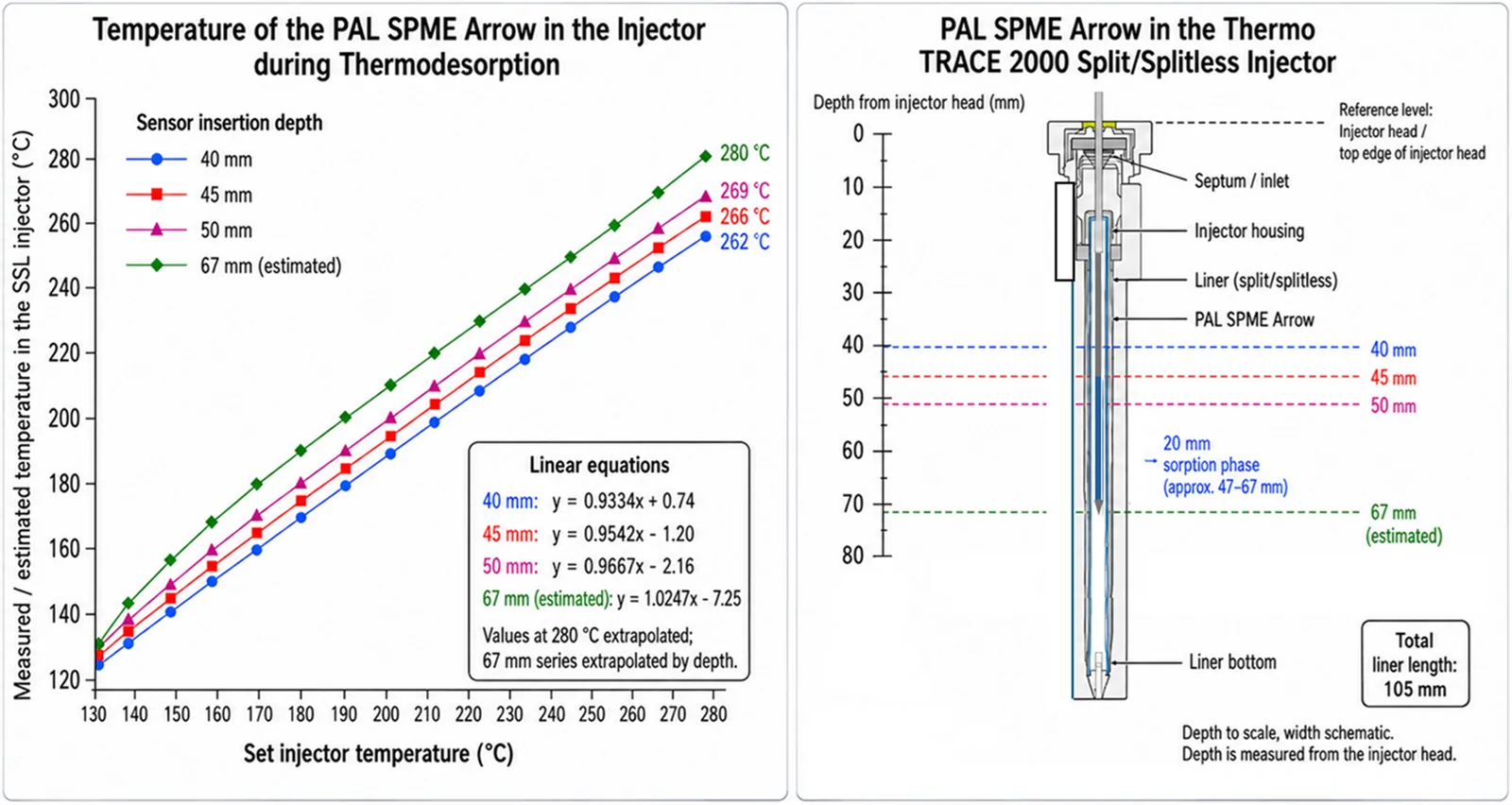
The left panel shows the measured temperature in the SSL GC injector as a function of injector set temperature at thermocouple probe depth of 40, 45, and 50 mm from a former liquid injection study (see Table S1). Last points were interpolated represent values estimated by linear extrapolation from the measured range of 130–250 °C. In the right panel a scheme of the split/splitless injector with SPME Arrow in relation to injection depth is shown

Desorption-and-transfer profiles were recorded according to the general approach described previously [16, 17]. Measurements were performed using a TRACE™ GC Ultra gas chromatograph coupled to a DSQ™ mass spectrometer (Thermo Electron Corporation, Austin, USA) equipped with a split/splitless injector (see Fig. 1). SPME Arrow injections were carried out with a PAL HTX-xt autosampler (CTC Analytics AG, Zwingen, Switzerland). The injector temperature was maintained at 280 °C throughout all experiments.

Carrier-gas flow rates of 0.8, 1.6, and 2.4 mL min⁻^1^ were investigated under splitless conditions. Preliminary experiments indicated that higher carrier-gas flow rates would have been desirable from a methodological perspective. However, flow rates above the investigated range occasionally caused uncontrolled split-valve opening, most likely due to pressure disturbances during insertion of the SPME Arrow into the injector. Because this effect occurred irregularly and would have compromised comparability, higher flow rates were not included in the final evaluation.

For the present study, the analytical column was replaced by a short capillary connection between the injector and the mass spectrometer to minimize chromatographic delay and allow direct recording of desorption-and-transfer profiles. A 1 m × 0.10 mm i.d. deactivated fused-silica capillary was used. Before recording desorption-and-transfer profiles, the applied carrier-gas flow rates were manually verified by a Flow Meter (Restek, Bad Homburg, Germany). These measurements served only to confirm the trueness of the set flow rates and are not shown.

The mass spectrometer started data acquisition simultaneously with SPME Arrow injection. Data were acquired in full-scan mode over an m/z range of 70–150 at a scan rate of 5.5 scans s⁻^1^. The ion source and transfer line temperatures were both set to 250 °C. To ensure comparability, all experiments were performed using a freshly cleaned ion source and the same tuning file throughout the measurement series.

All GC runs were carried out isothermally at 100 °C. Two injector liner geometries were investigated. Liner L1 was a commercially available 2.0 mm i.d. splitless liner (Restek, Bad Homburg, Germany). Liner L2 was a straight 1.2 mm i.d. liner manufactured in the glassblowing workshop of the University of Duisburg-Essen and deactivated according to procedures described by Grob and co-workers [19, 20].

### Evaluation of desorption-and-transfer profiles

**Fig. 2:**
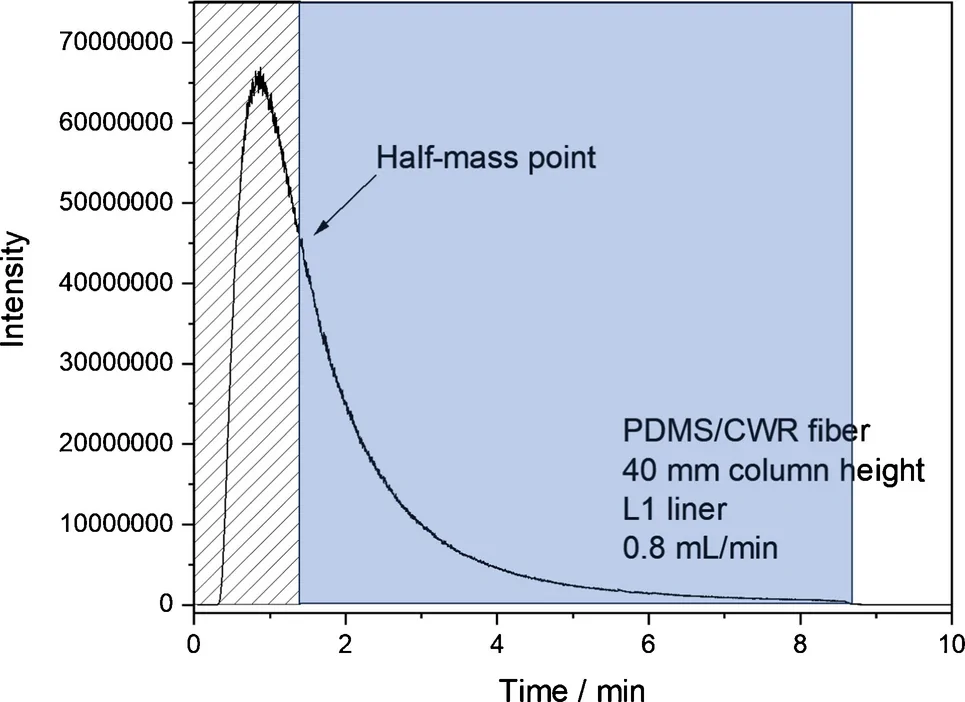
Representative desorption-and-transfer profile of 1-methylnaphthalene. The half-mass point corresponds to the time at which 50% of the total integrated signal has been detected

Desorption-and-transfer profiles were evaluated using XCalibur and OriginPro 2018b. For each measurement, the extracted ion chromatogram of 1-methylnaphthalene at m/z 142 was exported and integrated over a time interval from 0 to 10 min. The resulting area was defined as the total peak area and used as a measure of the amount of analyte transferred from the SPME Arrow coating through the injector to the detector (see Fig. 2).

The half-mass point, *t*_1/2_, was used as a kinetic descriptor. For each profile, the integration interval was iteratively shortened until the integrated area corresponded to 50% of the total peak area obtained between 0 and 10 min. The corresponding time was defined as *t*_1/2_. Lower *t*_1/2_ values indicate faster desorption and/or transfer. Following Bi and co-workers, twice the half-mass point was used as an operational comparative estimate of the characteristic desorption-and-transfer time under the respective conditions [21].

All measurements were performed in triplicate. Mean values were used for comparison of injection conditions.

For a 1 m × 0.10 mm i.d. deactivated fused-silica transfer capillary, the internal volume is approximately 7.85 µL. At a carrier-gas flow rate of 1.1 mL min⁻^1^, this corresponds to a theoretical transit time of only 0.43 s. This time is negligible compared with the experimentally observed half-mass points of approximately 0.4–1.4 min. Thus, the recorded desorption-and-transfer profiles are not determined by the hold-up time of the transfer capillary but by analyte desorption from the SPME Arrow coating and subsequent transfer through the injector.

### Calculation of relative desorption-and-transfer response

Because the two SPME Arrow coatings differ in sorptive properties and absolute response, normalization was performed separately for PDMS/CWR and PDMS/DVB. For each coating, the highest mean total peak area observed under the investigated conditions was set to 100%, and all other conditions were expressed relative to this value:

1 $$RT_{rel}\left(\%\right)=\frac{A_i}{A_{max}}'100$$

where ${RT}_{rel}$ is the relative desorption-and-transfer response, ${A}_{i}$ is the mean total peak area obtained under injection condition i, and ${A}_{max}$ is the highest mean total peak area observed for the respective coating. For PDMS/CWR, the maximum total peak area was obtained using the L2 liner at 40 mm column insertion depth and 1.6 mL min⁻^1^ carrier-gas flow. This condition was defined as 100% relative desorption-and-transfer response. For PDMS/DVB, the maximum total peak area was obtained using the L1 liner at 64 mm column insertion depth and 2.4 mL min⁻^1^ carrier-gas flow, which was defined as 100% relative desorption-and-transfer response.

This normalization allows the comparison of injection conditions within each coating while avoiding superposition of injection efficiency with coating-dependent extraction capacity, morphology, or analyte-coating interactions.

### Supporting information

The Supporting Information consists of three parts: A), B), and C). Supplementary part A) provides the temperature profile of the split/splitless injector as well as the applied method parameters (see Table S1 to S3).

Part B) contains the theoretical calculation of the breakthrough or transit time through the 1 m × 0.10 mm i.d. deactivated fused-silica transfer capillary. The internal capillary volume and the corresponding transit time at a carrier-gas flow rate of 1.1 mL min⁻^1^ are calculated to demonstrate that the transfer-capillary hold-up time is negligible compared with the experimentally observed half-mass points.

Part C) provides a simplified numerical assessment of the SPME Arrow liner geometry. This assessment includes the calculation of annular cross-sectional areas, apparent carrier-gas velocities, residence-time estimates, Reynolds numbers, steady-state pressure-drop estimates, and the sensitivity of the narrow annular gap to SPME Arrow eccentricity. The calculation compares the 2.0 mm i.d. L1 liner with the 1.2 mm i.d. L2 liner for a 1.1 mm SPME Arrow. It is intended as a comparative geometrical and pneumatic assessment and should not be interpreted as a validated CFD simulation or as a description of the transient pressure pulse during Arrow insertion. Supporting Information also includes the R script used to generate the supplementary calculations, tables, and figures.

## Results and discussion

### General considerations on injector geometry and carrier-gas velocity

Although the injector set temperature was 280 °C, a representative temperature of 273 °C was used for the simplified calculations. This value corresponds to the estimated mean temperature over the axial region occupied by the 20 mm SPME Arrow coating. It was derived from the previously determined position-dependent injector-temperature profile shown in Fig. 1. The value should therefore not be interpreted as the injector set temperature or as a direct measurement of the coating-surface temperature with the Arrow inserted [16, 17, 22, 23]. This takes account of the fact that the temperature is not uniform throughout the injector, so the position of the fiber within the injector can influence the desorption [24]. The SPME Arrow was inserted to the maximum mechanically accessible depth of 70 mm used in the present setup. Previous axial temperature measurements in the same SSL injector suggested that this position corresponds to a comparatively hot region of the liner. However, the actual coating-surface temperature was not experimentally determined with the Arrow inserted because of the narrow geometry of the injector.

The deactivated transfer capillary was inserted either 40 or 64 mm into the injector, corresponding to column positions used for splitless configurations. Its inner diameter was not varied, since wider deactivated transfer capillaries are typically preferred for the investigated setup [23, 25].

A central challenge in SPME Arrow method development is the selection of a suitable liner. For conventional SPME, narrow liners are recommended because they increase carrier-gas velocity around the fiber, reduce dead volume, improve heat transfer, and accelerate analyte transport. For SPME Arrow, the larger outer diameter restricts the choice of compatible liners and changes the free cross-sectional area available for gas flow.

Assuming laminar flow, the linear carrier-gas velocity u in the annular space between liner wall and fiber surface can be approximated by:

2 $$u=\frac F{{\displaystyle\frac \pi4}\left(d_{liner}^2-d_{fiber}^2\right)}$$

where F is the carrier-gas flow rate, ${d}_{liner}$ is the liner inner diameter, and ${d}_{fiber}$ is the fiber outer diameter. Carrier-gas flow affects the transfer of desorbed analytes through the GC inlet by controlling their residence time in the liner and the width of the injected analyte plug. Higher carrier-gas flow rates, or higher local linear gas velocities, shorten the residence time of analytes in the hot inlet and can promote faster transport toward the column entrance. This may reduce transfer-related band broadening, which is particularly relevant for SPME because no solvent focusing occurs during injection. In addition, shorter residence times can reduce the opportunity for adsorption, re-adsorption, or other interactions with inlet surfaces. Under splitless conditions, the transfer process is especially sensitive to liner geometry, liner volume, carrier-gas flow, purge timing, inlet temperature, and the initial oven temperature, all of which influence how efficiently the desorbed analyte is introduced into the column.

**Fig. 3:**
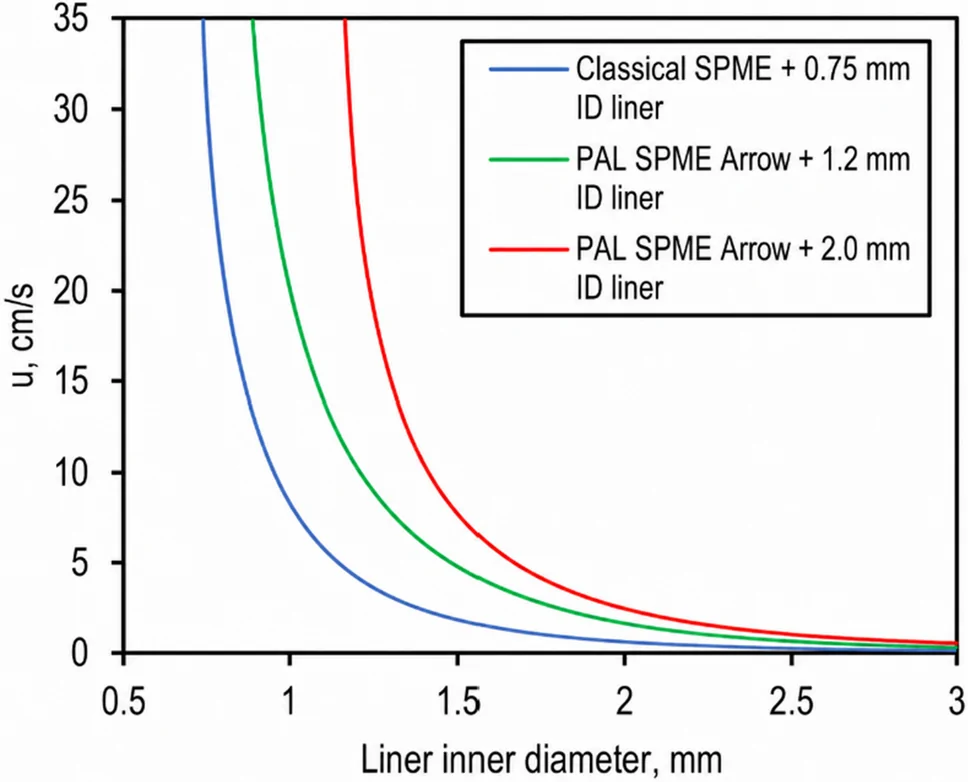
Calculated apparent linear carrier-gas velocities obtained from Eq. (2) for a conventional SPME fiber in a 0.75 mm i.d. liner and for a 1.1 mm SPME Arrow in 2.0 mm and 1.2 mm i.d. liners. The values are theoretical estimates used for geometrical comparison and do not represent experimentally measured local gas velocities inside the injector

Compared with a conventional SPME fiber in a 0.75 mm i.d. liner, a PAL SPME Arrow in a 2.0 mm i.d. liner has a substantially lower apparent carrier-gas velocity at the same volumetric flow rate. This may increase analyte residence time and slow transfer through the injector. Whether this also affects the total transferred analyte amount depends on coating-specific desorption kinetics, surface interactions, carrier-gas flow, and column positioning and therefore cannot be predicted from liner geometry alone. Therefore, the narrow self-made 1.2 mm i.d. L2 liner was compared with the commercially available 2.0 mm i.d. L1 liner (see Fig. 3). The values shown in Fig. 3 were calculated from Eq. (2) and therefore represent apparent theoretical carrier-gas velocities. They are intended for comparative assessment of the investigated liner geometries and do not represent experimentally measured local velocities inside the injector.

It has to be noted that Eq. 2 provides an apparent theoretical carrier-gas velocity based on the free annular cross-sectional area between the liner wall and the SPME Arrow surface. The calculation assumes an idealized concentric annular geometry and does not account for Arrow eccentricity, local velocity gradients, thermal expansion effects, temperature gradients, or transient pressure disturbances during Arrow insertion. Therefore, the calculated values are used only for comparative interpretation of the investigated liner geometries and should not be interpreted as true local carrier-gas velocities inside the injector. The observed trends should not be interpreted solely on the basis of the idealized apparent carrier-gas velocity. Fiber eccentricity, local velocity gradients, carrier-gas thermal expansion, pressure disturbances during Arrow insertion, and coating-dependent desorption-and-transfer kinetics may all contribute to the experimentally observed desorption-and-transfer behavior.

The simplified numerical assessment provided discussion which further illustrates the pronounced difference between the two liner geometries (see Tables S5 to S9 as well as Figures S1 to S4 in Supplementary Note C). For a 1.1 mm SPME Arrow, the 1.2 mm i.d. L2 liner reduces the free annular cross-sectional area by approximately a factor of 12 compared with the 2.0 mm i.d. L1 liner. Over the full liner length of 105 mm and the investigated carrier-gas flow range of 0.8–2.4 mL min⁻^1^, the calculated steady-state pressure drop ranges from approximately 2.0 to 6.1 kPa for L2, compared with approximately 2.1 to 6.2 Pa for L1. Thus, the simplified calculation indicates that the narrow L2 geometry is substantially more pneumatically restrictive than L1. However, these values were calculated assuming steady-state concentric laminar annular flow and should not be interpreted as a quantitative description of the transient pressure pulse occurring during SPME Arrow insertion.

### Influence of liner geometry, carrier-gas flow, and column position on injection efficiency

The total peak areas obtained for PDMS/CWR and PDMS/DVB show that SPME Arrow injection is strongly affected by liner geometry, carrier-gas flow rate, column position, and coating chemistry. Since the total peak area reflects the amount of 1-methylnaphthalene reaching the detector after desorption and transfer through the injector and the very short GC column, it provides a practical measure of overall injection efficiency. The two coatings, however, showed markedly different behavior.

**Fig. 4:**
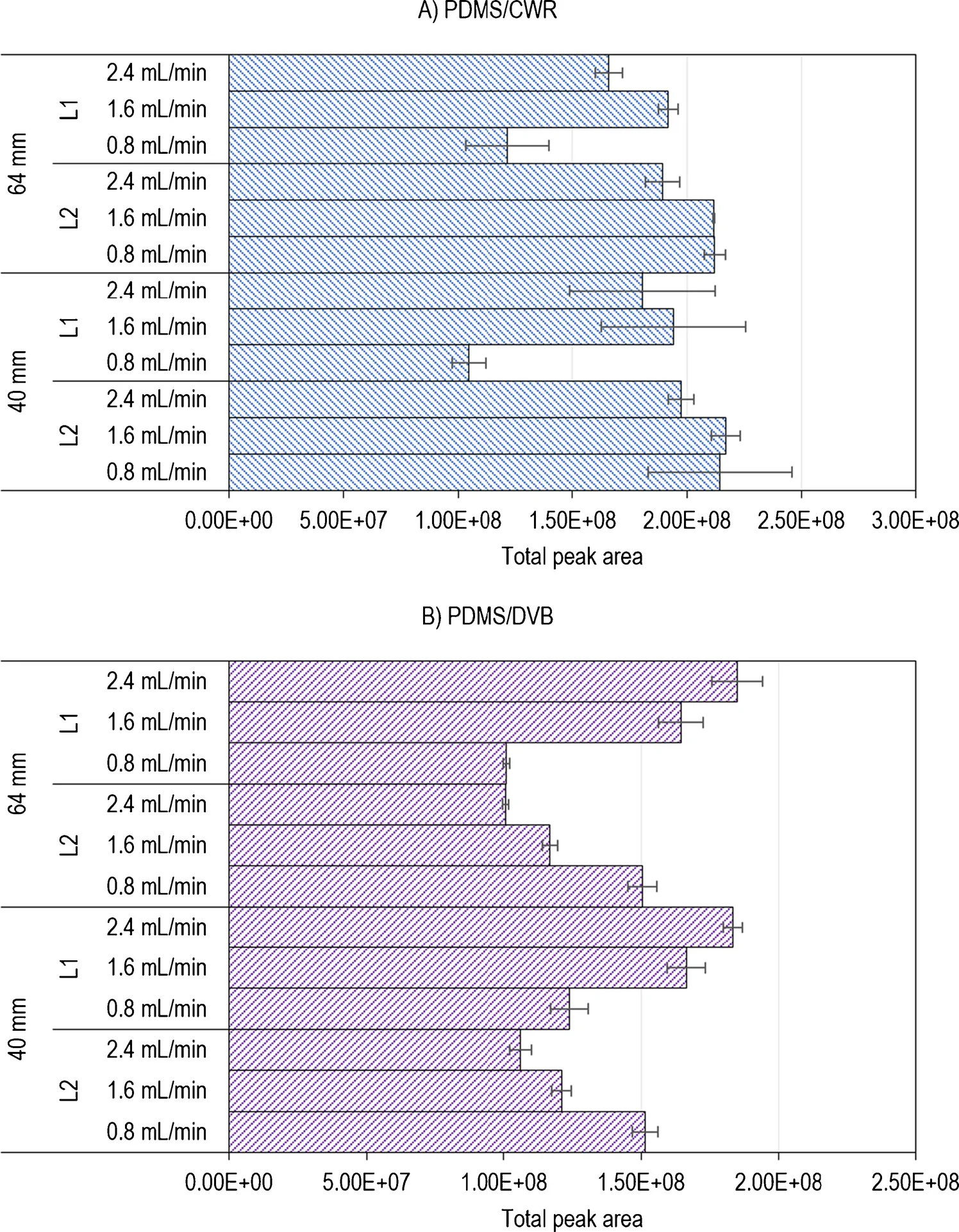
Total area under the curve obtained under different injection conditions with **A** PDMS/CWR and **B** PDMS/DVB fibers

For the PDMS/CWR Arrow, the effect of liner geometry was pronounced. The narrow L2 liner provided consistently high relative desorption-and-transfer response under almost all investigated conditions. At 40 mm column insertion depth, relative desorption-and-transfer response of 98.85%, 100.0%, and 91.04% were obtained at carrier-gas flow rates of 0.8, 1.6, and 2.4 mL min⁻^1^, respectively. At 64 mm, corresponding values were 97.76%, 97.63%, and 87.29%. Thus, the narrow liner enabled efficient analyte transfer over a broad range of flow rates (see Fig. 4).

In contrast, the wider L1 liner resulted in lower and more flow-dependent injection efficiencies for PDMS/CWR. At 40 mm and 0.8 mL min⁻^1^, the relative desorption-and-transfer response was only 48.21%, compared with 98.85% for the L2 liner under otherwise identical conditions. At 64 mm and 0.8 mL min⁻^1^, L1 reached 56.06%, again substantially lower than L2. Increasing the carrier-gas flow improved the response in L1, but the wider liner did not provide the same consistently high injection efficiencies as L2.

At a given volumetric flow rate, the wider annular space results in a lower apparent carrier-gas velocity and a longer estimated residence time. For PDMS/CWR, this may increase the opportunity for dilution, adsorption, re-adsorption, or delayed transfer and may therefore contribute to the lower total peak areas observed at low carrier-gas flow. However, the contrasting results obtained for PDMS/DVB demonstrate that liner geometry alone does not determine the overall transferred amount. At a given volumetric flow rate, the wider annular space results in a lower local linear carrier-gas velocity. The larger annular volume of the L1 liner and the correspondingly lower apparent carrier-gas velocity are consistent with longer gas-phase residence times and slower convective transport. However, the experimental data do not permit the observed differences in total peak area and transfer kinetics to be attributed to a single mechanism. In addition to gas-phase transport, the results may reflect coupled effects of coating-specific desorption kinetics, diffusion and mass transfer within the coating, spatial and transient temperature variations, local flow trajectories, Arrow eccentricity, surface interactions, pressure disturbances during insertion, and analyte transfer into the capillary entrance. The simplified geometrical calculations show substantial differences in apparent carrier-gas velocity and estimated residence time between the two liner geometries. These differences are compatible with an influence of liner geometry on the measured desorption-and-transfer profiles. Nevertheless, the present experiments do not resolve the underlying mechanism. The observed coating-dependent responses probably result from several interacting thermal, fluid-dynamic, and mass-transfer processes, and should not be interpreted solely in terms of apparent gas velocity or inlet dilution.

The lower apparent carrier-gas velocity and longer estimated residence time in the L1 liner are consistent with the slower transfer observed for PDMS/CWR. However, these calculations do not establish the mechanism responsible for the differences in total peak area. The lower half-mass points obtained with L2 demonstrate faster overall desorption-and-transfer, while the contribution of convective transport relative to thermal and coating-specific effects cannot be resolved.

**Fig. 5:**
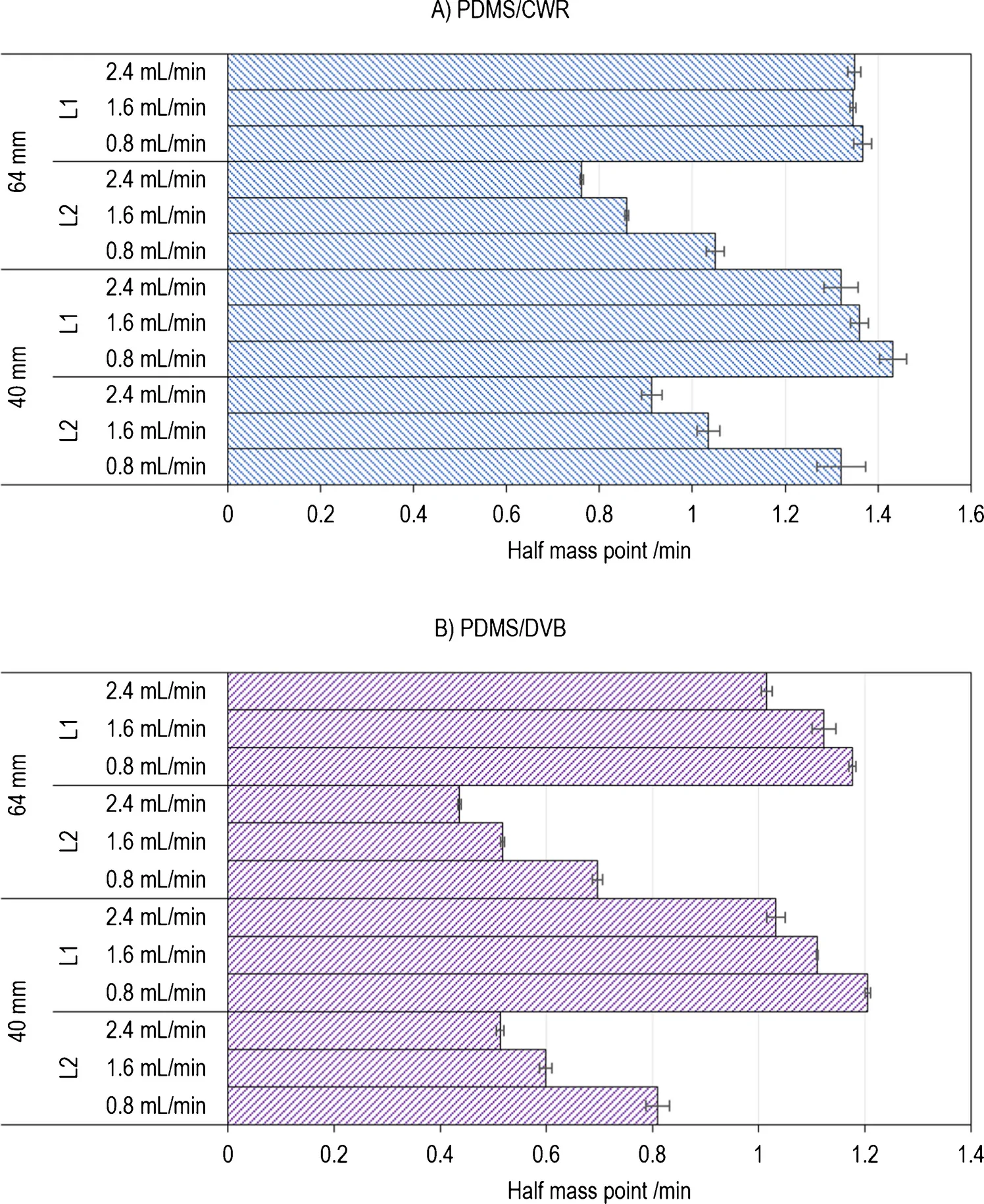
Half mass point under the desorption curve obtained under different injection conditions with **A** PDMS/CWR and **B** PDMS/DVB fibers In both cases it is evident that the narrower, L2 liner results in much quicker transfer of 1-methylnaphthalene onto the column. Furthermore, the dependency of the flowrates is only pronounced if this, narrower liner is used.

The half-mass points support this interpretation. For PDMS/CWR, L2 produced faster transfer than L1. At 40 mm, the half-mass point decreased from 1.32 min at 0.8 mL min⁻^1^ to 1.03 min at 1.6 mL min⁻^1^ and 0.91 min at 2.4 mL min⁻^1^. At 64 mm, the same trend was more pronounced, with values of 1.04, 0.85, and 0.76 min. In L1, half-mass points remained comparatively high and changed only slightly with flow rate. This indicates that increasing carrier-gas flow in the wide liner improved the total transferred amount to some extent but did not accelerate transfer as effectively as in the narrow liner (see Fig. 5).

For PDMS/CWR, the injection process therefore appears to be strongly transfer-limited when a wide liner is used. The carbon-containing phase may retain 1-methylnaphthalene more strongly, making efficient heat transfer and rapid removal of desorbed analyte especially important. Under these conditions, reducing liner volume and increasing carrier-gas velocity are beneficial for both transfer rate and total transferred amount.

PDMS/DVB showed a different response. Here, the highest desorption-and-transfer responses were obtained with the wider L1 liner at elevated carrier-gas flow rates. At 40 mm, L1 yielded desorption-and-transfer responses of 67.05%, 90.05%, and 99.21% at 0.8, 1.6, and 2.4 mL min⁻^1^, respectively. At 64 mm, the corresponding values were 54.68%, 88.96%, and 100.00%. Thus, for PDMS/DVB, L1 at 2.4 mL min⁻^1^ provided the highest total peak areas at both column insertion depths.

By contrast, the L2 liner did not improve total transferred amount for PDMS/DVB. At 40 mm, desorption-and-transfer responses decreased from 81.89% at 0.8 mL min⁻^1^ to 65.52% at 1.6 mL min⁻^1^ and 57.45% at 2.4 mL min⁻^1^. At 64 mm, the same trend was observed, with values of 81.40%, 63.23%, and 54.51%. This trend is opposite to what would be expected if injection efficiency were governed only by linear carrier-gas velocity.

The half-mass points clarify this apparent contradiction. With L2, PDMS/DVB showed rapid transfer, and *t*_1/2_ decreased strongly with increasing flow rate. At 40 mm, *t*_1/2_ decreased from 0.8098 min to 0.5985 and 0.5130 min. At 64 mm, even lower values of 0.6963, 0.5171, and 0.4364 min were obtained. Thus, L2 clearly accelerated transfer. However, this faster transfer was accompanied by lower total peak areas, especially at higher flow rates. The fastest transfer condition was therefore not the most efficient in terms of analyte recovery (see Table 1).

**Table 1:** Total peak areas, relative desorption-and-transfer response, half-mass points, and standard deviations for all investigated combinations of coating, column insertion depth, liner geometry, and carrier-gas flow rate

Fiber	Position	Liner	Flow in mL min⁻^1^	Total peak area	Relative RT in %	Area SD	Relative RT SD in %	Half-mass point in min	Half-mass SD in min
PDMS/CWR	40 mm	L2	0.8	214,386,000	98.85	31,328,250	14.44	1.3208	0.0524
		L2	1.6	216,887,500	100.00	6,204,862	2.86	1.0349	0.0242
		L2	2.4	197,461,500	91.04	5,617,352	2.59	0.9124	0.0220
		L1	0.8	104,565,125	48.21	7,381,440	3.40	1.4323	0.0290
		L1	1.6	194,162,500	89.52	31,497,586	14.52	1.3598	0.0195
		L1	2.4	180,587,750	83.26	31,699,235	14.62	1.3201	0.0370
	64 mm	L2	0.8	212,022,750	97.76	4,633,328	2.14	1.0493	0.0193
		L2	1.6	211,741,667	97.63	371,947	0.17	0.8586	0.0042
		L2	2.4	189,327,750	87.29	7,423,503	3.42	0.7613	0.0034
		L1	0.8	121,591,500	56.06	18,309,414	8.44	1.3666	0.0196
		L1	1.6	191,781,750	88.42	4,217,360	1.94	1.3461	0.0065
		L1	2.4	165,839,750	76.46	5,932,588	2.74	1.3488	0.0135
PDMS/DVB	40 mm	L2	0.8	151,367,500	81.89	4,668,432	2.53	0.8098	0.0219
		L2	1.6	121,096,000	65.52	3,543,358	1.92	0.5985	0.0117
		L2	2.4	106,194,667	57.45	3,902,939	2.11	0.5130	0.0072
		L1	0.8	123,938,000	67.05	6,761,355	3.66	1.2055	0.0049
		L1	1.6	166,452,667	90.05	6,897,784	3.73	1.1107	0.0015
		L1	2.4	183,369,000	99.21	3,491,693	1.89	1.0325	0.0177
	64 mm	L2	0.8	150,457,750	81.40	5,184,234	2.80	0.6963	0.0097
		L2	1.6	116,867,250	63.23	2,768,667	1.50	0.5171	0.0035
		L2	2.4	100,757,450	54.51	1,087,527	0.59	0.4364	0.0032
		L1	0.8	101,062,600	54.68	1,167,062	0.63	1.1765	0.0069
		L1	1.6	164,436,333	88.96	8,133,783	4.40	1.1231	0.0224
		L1	2.4	184,835,750	100.00	9,232,917	5.00	1.0150	0.0101

For some parameter combinations, relatively high standard deviations were observed for the integrated peak areas. Since this was not necessarily accompanied by similarly high variability in the half-mass points, the temporal desorption-and-transfer behavior appears to be more reproducible than the total recovered signal. The variance is therefore likely related to replicate-to-replicate differences in analyte loading, SPME Arrow positioning, local thermal contact, carrier-gas flow trajectories, and coating-internal mass-transfer effects.

This behavior suggests that the limiting step differs between coatings. PDMS/DVB may release 1-methylnaphthalene more rapidly than PDMS/CWR at the applied injector temperature. If desorption is already fast, further increasing local carrier-gas velocity in the narrow liner may not improve total transferred amount. Instead, reduced residence time, altered flow trajectories, or transient inlet pressure effects may reduce efficient capture by the column inlet. The higher total peak areas obtained with the wider L1 liner at elevated flow rates cannot be assigned to a single mechanism. The larger annular flow area and lower calculated steady-state flow resistance may contribute, but transient pressure behavior and local flow conditions were not experimentally resolved.

Column insertion depth had a smaller effect than liner geometry and coating type. For PDMS/CWR with L2, both 40 and 64 mm provided high desorption-and-transfer responses, although the maximum was observed at 40 mm and 1.6 mL min⁻^1^. Differences between the two investigated capillary insertion positions were comparatively small and did not show a consistent trend across coatings, liner geometries, and carrier-gas flow rates. For PDMS/DVB, similarly high L1 responses were obtained at both capillary insertion positions at 2.4 mL min⁻^1^.

Overall, total peak area and half-mass point describe complementary aspects of SPME Arrow injection. For PDMS/CWR, L2 improved both quantitative transfer and transfer kinetics. For PDMS/DVB, L2 accelerated transfer but did not maximize total analyte recovery. Thus, injection parameters optimized for one SPME Arrow phase cannot be transferred directly to another without experimental verification.

The narrow L2 liner provides only a small annular clearance around the 1.1 mm SPME Arrow. Although this geometry is physically compatible, the small annular gap may induce transient pressure disturbances during Arrow insertion because the Arrow temporarily displaces gas in a piston-like manner. These transient pressure pulses are distinct from the steady-state apparent carrier-gas velocities calculated from the annular cross-sectional area. In the present work, carrier-gas flow rates above the investigated range occasionally can cause uncontrolled split-valve opening, most likely due to such transient pressure disturbances. Therefore, higher flow rates were excluded from the final evaluation. For routine automated and high-throughput applications, the use of very narrow liners may require careful verification of inlet-pressure stability, autosampler alignment, septum integrity, SPME Arrow alignment, and long-term robustness. Although the L2 liner can improve apparent transfer and, depending on coating chemistry, the relative desorption-and-transfer response, its narrow annular gap may provide lower mechanical and pneumatic tolerance than a wider liner. Septum lifetime and long-term robustness were not systematically investigated in the present study; therefore, no quantitative conclusions regarding septum lifetime or the maximum number of consecutive injections with the L2 liner can be drawn from the present data. These aspects should be evaluated separately before the narrow liner is applied in extended routine sequences.

Comparison of the total peak areas and half-mass points shows that SPME Arrow injection conditions cannot be selected solely by minimizing the half-mass point or maximizing the apparent carrier-gas velocity. Instead, kinetic and quantitative descriptors should be considered together. The condition providing the fastest apparent transfer does not necessarily provide the highest transferred analyte amount. To provide a concise practical overview, the experimentally preferred conditions for either maximum relative desorption-and-transfer response or fastest apparent transfer are summarized in Table 2. These results further demonstrate that the preferred condition depends on the analytical objective and coating chemistry.

**Table 2:** Summary of experimentally preferred injection conditions for the investigated SPME Arrow coatings under the conditions of this study

Coating	Analytical objective	Preferred liner	Flow rate	Capillary insertion depth	Experimental observation
PDMS/CWR	Maximum relative desorption-and-transfer response	L2, 1.2 mm i.d.	1.6 mL min⁻^1^	40 mm	Highest total peak area, defined as 100% relative desorption-and-transfer response
PDMS/CWR	Fast transfer with high response	L2, 1.2 mm i.d.	1.6–2.4 mL min⁻^1^	40 or 64 mm	Lower half-mass points while maintaining high relative desorption-and-transfer response
PDMS/DVB	Maximum relative desorption-and-transfer response	L1, 2.0 mm i.d.	2.4 mL min⁻^1^	64 mm	Highest total peak area, defined as 100% relative desorption-and-transfer response
PDMS/DVB	Fastest apparent transfer	L2, 1.2 mm i.d.	2.4 mL min⁻^1^	64 mm	Lowest half-mass point, but with reduced total peak area

The preferred condition depends on whether maximum transferred analyte amount or fastest apparent transfer is prioritized. For quantitative applications, maximum total peak area should generally be prioritized, whereas the half-mass point provides complementary information on transfer kinetics and potential peak broadening. The conditions summarized here refer specifically to the investigated coatings, model analyte, and experimental setup and should not be interpreted as universally optimal SPME Arrow injection conditions

## Conclusions

This study demonstrates that SPME Arrow-GC-MS injection is governed by a combined desorption-and-transfer process rather than by thermal desorption alone. Total peak area and half-mass point provided complementary information: the total peak area reflected the amount of analyte transferred to the analytical system, whereas the half-mass point described the rate of transfer.

The results show that injection parameters optimized for one SPME Arrow coating cannot necessarily be transferred to another. Liner geometry, carrier-gas flow rate, column insertion depth, and coating chemistry must be considered together. For method development, both total transferred amount and transfer kinetics should be evaluated, since the fastest transfer condition is not always the condition that provides the highest analyte recovery. Injection conditions optimized for one SPME Arrow coating cannot necessarily be transferred directly to another coating. Coating-specific optimization is recommended because maximum recovery and fastest transfer may occur under different injector configurations.

The coating-dependent behavior observed here is consistent with the general understanding that microextraction sorbents differ in thermodynamic and kinetic sorption parameters. Although the present setup was not designed to determine sorption enthalpies or partitioning constants, the different responses of PDMS/CWR and PDMS/DVB indicate that coating-specific analyte-phase interactions contribute substantially to the observed desorption-and-transfer behavior. Future experiments at several injector temperatures and constant flow/geometry conditions could provide apparent desorption or transfer activation energies and help distinguish thermodynamic coating effects from injector-related transport limitations.

Overall, this work highlights the importance of systematic optimization of the injection step in SPME Arrow analysis. Purpose-designed Arrow-compatible liners that reduce dead volume while maintaining stable inlet flow and efficient analyte guidance toward the column entrance may further improve sensitivity, repeatability, and robustness of SPME Arrow-GC-MS methods.

## Supplementary Information

Below is the link to the electronic supplementary material.

ESM 1 (DOCX 175 KB)

## Data Availability

Data are available from the authors by request.

## Declarations

### Statement of AI use

The AI-based language model GPT-5 (OpenAI, San Francisco, USA) was used. The tool served solely as an assistance aid, structuring of text drafts, and code generation for figures. All contents and figures were reviewed, revised, and are fully the responsibility of the authors. The use of AI had no influence on the research design, data collection, or interpretation of the results.
