# Supplementary material for: Determination of the optimal injection parameters for the SPME Arrow analysis of VOCs
Source: Anal Bioanal Chem. 2026 Aug 31;418(22):7567–78. doi: 10.1007/s00216-026-06757-y (PMC13736470; doi:10.1007/s00216-026-06757-y)
Supplement: Supplementary file 1 [file 216_2026_6757_MOESM1_ESM.docx]

**Supplementary Material for Determination of the optimal injection parameters for the SPME Arrow analysis of VOCs**

Nenad Stojanović^1^, Maik A. Jochmann^1^, Torsten C. Schmidt^1, 3, 4^

^1^Instrumental Analytical Chemistry, University of Duisburg-Essen, Universitätsstrasse 5, 45141 Essen, Germany

^3^Centre for Water and Environmental Research, University of Duisburg-Essen, Universitätsstrasse 5, 45141 Essen, Germany

^4^IWW Water Centre, Moritzstrasse 26, 45476 Mülheim an der Ruhr, Germany

# A) Temperature profile of the split/splitless injector and method parameters

In Table 1, the temperature profile of a split/splitless (SSL) injector belonging to a Trace GC Ultra, Thermo Electron Corporation, Bremen, Germany)is shown. Injection depth were 0 - 50 mm from septum; in 5 mm steps. The temperatures were set from 130 - 250°C in 10°C steps. A thermometer thermocouple sensor (Eurotherm, Chessell GmbH, VA, USA) was introduced into the injector at different depths and the measured temperature was observed. Depending on the time, which the injectors and the thermometer sensor needed to reach a constant value, results were recorded after 5 min (SSL), respectively.

**Table S1.** Measured temperatures in °C in the SSL injector at certain set temperatures and certain depths.

| **Set temperature** | **Depth [mm]** | | | | | | | | | | |
| --- | --- | --- | --- | --- | --- | --- | --- | --- | --- | --- | --- |
| **in [°C]** | **0** | **5** | **10** | **15** | **20** | **25** | **30** | **35** | **40** | **45** | **50** |
| **130** | 105.8 | 110.9 | 113.5 | 116.4 | 117.3 | 115.5 | 118.9 | 117.9 | 122.9 | 123.9 | 123.1 |
| **140** | 109.7 | 119.2 | 121.9 | 122.3 | 123.7 | 124.4 | 126.1 | 127.8 | 131.3 | 132.3 | 133.4 |
| **150** | 116.6 | 127.4 | 130.4 | 130.7 | 131.9 | 133.2 | 134.5 | 136.9 | 140.6 | 141.7 | 143.1 |
| **160** | 123.6 | 135.5 | 138.6 | 139.8 | 141.0 | 142.2 | 144.2 | 146.4 | 150.1 | 151.3 | 152.7 |
| **170** | 131.3 | 144.1 | 147.5 | 148.4 | 149.0 | 151.7 | 153.1 | 155.4 | 158.9 | 160.7 | 162.2 |
| **180** | 139.0 | 153.0 | 156.2 | 158.0 | 158.2 | 159.8 | 162.4 | 164.7 | 168.6 | 170.2 | 171.9 |
| **190** | 146.7 | 161.3 | 165.0 | 166.4 | 167.3 | 168.4 | 170.8 | 174.1 | 177.6 | 179.9 | 181.3 |
| **200** | 153.7 | 169.8 | 172.4 | 175.6 | 175.9 | 177.6 | 180.0 | 182.7 | 187.3 | 189.5 | 190.9 |
| **210** | 162.2 | 178.8 | 181.7 | 184.4 | 185.2 | 186.3 | 189.2 | 192.2 | 196.8 | 198.9 | 200.9 |
| **220** | 169.9 | 187.6 | 190.8 | 192.5 | 193.3 | 195.0 | 198.2 | 201.4 | 206.4 | 208.6 | 210.7 |
| **230** | 177.0 | 196.4 | 199.3 | 201.6 | 202.7 | 204.2 | 199.8 | 211.0 | 216.0 | 218.5 | 220.1 |
| **240** | 184.9 | 205.7 | 207.8 | 210.9 | 212.1 | 213.6 | 207.7 | 221.7 | 223.9 | 228.0 | 229.8 |
| **250** | 192.9 | 215.4 | 217.1 | 219.3 | 220.3 | 222.0 | 217.1 | 228.9 | 234.7 | 237.8 | 239.6 |

**Table S2.** Method steps and parameters

| **Method step / parameter** | **Setting** |
| --- | --- |
| **GC system** | Thermo TRACE 2000 GC |
| **Injector** | Split/splitless injector |
| **Autosampler / tool** | PAL SPME Arrow |
| **Liner length** | 105 mm |
| **Depth reference point** | From the injector head  (0 mm = top edge of the injector head) |
| **Arrow insertion depth** | 70 mm |
| **Maximum PAL penetration depth** | 70 mm |
| **Sorption-phase length** | 20 mm |
| **Approximate sorption-phase position** | Approximately 47–67 mm |
| **Injector temperature set (Thermodesorption temperature set)** | 280 °C |
| **Injektor temperature at 47 mm depth (upper part of the Arrow coating)** | 266 °C |
| **Estimated temperature at 67 mm depth (lower part of the Arrow coating)** | ~280 °C  (Based on the depth extrapolation) |
| **Injection mode** | Splitless |
| **Thermodesorption time** | 10 min |
| **Splitless time** | 10 min |
| **Carrier-gas flow** | See in the paper |
|  |  |

**Table S3.** SPME Arrow dimensions and phase properties

| Coating / phase | Arrow OD | Phase thickness | Phase length | Analyte class / intended range |
| --- | --- | --- | --- | --- |
| ****PDMS/CWR**** | 1.1 mm | 120 µm | 20 mm | Highly volatile and low-molecular-weight compounds |
| ****PDMS/DVB**** | 1.1 mm | 120 µm | 20 mm | Aromatic and semi-volatile compounds |

# B) Theoretical Breakthrough Time of a Deactivated Transfer Capillary

The carrier-gas flow rate of 1.1 mL min⁻¹ is assumed, the theoretical transit time through the 1 m × 0.10 mm i.d. deactivated fused-silica transfer capillary can be estimated from the capillary volume and the volumetric flow rate.

**Table S4** Parameters used for the calculation of the breakthrough time.

| Parameter | Value |
| --- | --- |
| Capillary length, *L* | 1000 mm |
| Capillary inner diameter | 0.10 mm |
| Capillary radius, *r* | 0.05 mm |
| Carrier-gas flow rate, *Q* | 1.1 mL min⁻¹ = 1100 µL min⁻¹ |

**Calculation:**

*V = π · r² · L = π* × (0.05 mm)² × 1000 mm *=* 7.85 mm³ = 7.85 µL

*Q =* 1.1 mL min⁻¹ = 1100 µL min⁻¹

*t = V / Q =* (7.85 µL) / (1100 µL min⁻¹) = 0.00714 min = 0.43 s

At a carrier-gas flow rate of 1.1 mL min⁻¹, the theoretical breakthrough or transit time through the 1 m × 0.10 mm i.d. deactivated transfer capillary is approximately 0.43 s. This matches with the breakthrough time shown in Figure 2.

# C) Simplified numerical assessment of SPME Arrow liner geometry

This supplementary note combines the simplified numerical assessment of SPME Arrow liner geometry with the supplementary pressure-drop calculation. The calculation was performed for a 1.1 mm SPME Arrow operated in a 2.0 mm i.d. liner (L1) and a 1.2 mm i.d. liner (L2). It is intended only as comparative support for interpreting the experimental trends observed in the manuscript.

The calculation is not a validated CFD model. It does not resolve transient insertion effects, detailed injector temperature fields, local three-dimensional velocity profiles, coating-specific desorption kinetics, or active pressure-control behaviour of the instrument. In particular, the pressure-drop estimate describes steady-state concentric laminar annular flow and is not intended to describe the transient pressure pulse during SPME Arrow insertion.

Table S5. Parameters used for simplified numerical assessment.

| Parameter | Value |
| --- | --- |
| Carrier gas | Helium |
| SPME Arrow outer diameter used in model | 1.1 mm |
| Coating length | 20 mm |
| Wide liner L1 inner diameter | 2.0 mm |
| Narrow liner L2 inner diameter | 1.2 mm |
| Thermo Trace SSL liner length | 105 mm |
| Carrier-gas inlet temperature | 23 °C |
| Injector temperature | 273 °C |
| Thermal expansion factor *T_inj_/T_in_* | 1.844 |
| Helium density at 273 °C and 1 atm | 0.0893 kg m^-3^ |
| Helium dynamic viscosity at 273 °C | 2.97 x 10^-5^ Pa s |

The thermal expansion factor was calculated as $T_{\mathrm{injector}}/T_{\mathrm{inlet}}=546.15/296.15=1.844$. Helium density at 273 °C and 1 atm was estimated from the ideal gas law, $\rho=PM/(RT)$, giving $0.0893\text{ }kg\text{ }m^{-3}$. The dynamic viscosity of helium at 273 °C was estimated using Sutherland’s equation, giving $2.99\times{10}^{-5}\text{ }Pa\text{ }s$.

Sutherland-Gleichung:

$$\mu=\mu_{\mathrm{ref}}\left( \frac{T}{T_{\mathrm{ref}}} \right)^{3/2}\frac{T_{\mathrm{ref}}+S}{T+S}$$

with

$$\mu_{\mathrm{ref}}=1.864\times{10}^{-5}\text{ }Pa\text{ }s$$

$$T_{\mathrm{ref}}=273.15\text{ }K$$

$S=79.4\text{ }K$ *from https://doc.comsol.com/*

$$T=546.15\text{ }K$$

$$\mu=2.97\times{10}^{-5}\text{ }Pa\text{ }s$$

These values were used only for simplified comparative calculations and not as experimentally measured gas properties.

**Equations:**

Annular cross-sectional area: $A\text{ }=\text{ }\pi/4\text{ }\cdot\text{ }\left(D_{liner}^{2}\text{ }-\text{ }D_{Arrow}^{2}\right)$

Apparent linear carrier-gas velocity: $u_{app}\text{ }=\text{ }Q/A$

Thermal expansion correction: *Q_273_ = Q_23_ (T_inj_/T_in_)*, assuming ideal-gas behavior at constant pressure.

Residence-time estimate: *τ = L/u_app_*, calculated for the 20 mm coating length and for the 105 mm liner

length.

Hydraulic diameter for a concentric annulus: *D_h_ = D_liner_ - D_Arrow_*

Reynolds number: *Re = ρ u D_h_ /µ*

The steady-state pressure-drop estimate for concentric laminar annular flow was calculated from the analytical Hagen-Poiseuille solution for an annulus. This estimate was used only to compare the relative flow resistance of L1 and L2 and does not describe the transient pressure pulse during Arrow insertion.

Eccentricity sensitivity was evaluated using narrow-gap approximation. The concentric radial gap was defined as *c = (D_liner_ - D_Arrow_)/2*, and the lateral displacement of the Arrow from the liner centerline was denoted as *e*. The local minimum and maximum gap heights were therefore estimated as *h_min_ = c - e* and *h_max_ = c + e*. Since local hydraulic conductance scales approximately with h^3^, even modest eccentricity can produce pronounced local velocity non-uniformity. This approximation was used only to illustrate the potential magnitude of this effect and was not intended as a full eccentric annular-flow solution.

**Calculated geometry:**

Table S6. Geometrical parameters of the annular space around a 1.1 mm SPME Arrow.

| Geometry | Liner ID (mm) | SPME Arrow OD (mm) | Radial clearance (mm) | Annular area (mm²) | Hydraulic diameter (mm) | Area ratio vs L2 |
| --- | --- | --- | --- | --- | --- | --- |
| L1 wide liner | 2.000 | 1.100 | 0.450 | 2.191 | 0.900 | 12.13 |
| L2 narrow liner | 1.200 | 1.100 | 0.050 | 0.181 | 0.100 | 1.000 |

The narrow L2 liner reduces the annular free cross-section by a factor of approximately 12 relative to the 2.0 mm L1 liner. Consequently, the apparent linear carrier-gas velocity around the Arrow is approximately 12-fold higher in L2 at the same volumetric flow rate.

## Calculated apparent velocity and residence time:

Table S7. Apparent velocity and residence-time estimates.

| Geometry | Flow  (mL min^-1^) | *u_app_* at 23 °C (mm s^-1^) | u_app_ at 273 °C (mm s^-1^) | Residence time over 20 mm (s) | Residence time over 105 mm (s) |
| --- | --- | --- | --- | --- | --- |
| L1 wide liner | 0.800 | 6.085 | 11.22 | 1.782 | 9.357 |
| L1 wide liner | 1.600 | 12.17 | 22.44 | 0.891 | 4.679 |
| L1 wide liner | 2.400 | 18.25 | 33.66 | 0.594 | 3.119 |
| L2 narrow liner | 0.800 | 73.81 | 136.12 | 0.146 | 0.771 |
| L2 narrow liner | 1.600 | 147.6 | 272.2 | 0.073 | 0.386 |
| L2 narrow liner | 2.400 | 221.4 | 408.4 | 0.049 | 0.257 |

Figure S1. Calculated apparent linear carrier-gas velocities at injector temperature for the investigated liner geometries.

Figure S2. Estimated residence times over the 20 mm coating length. The logarithmic y-axis is used because of the large difference between the liner geometries.

## Laminarity and pressure-drop estimate:

Table S8. Reynolds numbers and steady-state pressure-drop estimates for concentric annular flow.

| Geometry | Flow (mL min-1) | Reynolds number at 273 °C | Estimated *Δp* over 20 mm (Pa) | Estimated *Δp* over 105 mm (Pa) |
| --- | --- | --- | --- | --- |
| L1 wide liner | 0.800 | 0.030 | 0.393 | 2.061 |
| L1 wide liner | 1.600 | 0.060 | 0.785 | 4.123 |
| L1 wide liner | 2.400 | 0.091 | 1.178 | 6.184 |
| L2 narrow liner | 0.800 | 0.041 | 388.1 | 2037 |
| L2 narrow liner | 1.600 | 0.081 | 776.1 | 4074 |
| L2 narrow liner | 2.400 | 0.123 | 1164 | 6112 |

The calculated Reynolds numbers are far below unity for both liner geometries. The simplified steady-state calculation therefore supports laminar-flow assumptions. However, the estimated pressure drop for the narrow L2 geometry is approximately three orders of magnitude higher than for L1. This does not describe the transient insertion event, but it supports the interpretation that the narrow annular geometry is substantially more pneumatically restrictive and more sensitive to disturbances during Arrow insertion.

Figure S3. Estimated steady-state pressure drop over the 105 mm liner length. The calculation assumes concentric laminar annular flow and is intended only for comparative interpretation.

## Sensitivity to fiber eccentricity:

Table S9. Narrow-gap eccentricity sensitivity based on local gap variation and approximate *h*^3^ conductance scaling.

| Geometry | Nominal radial clearance (µm) | Eccentricity e (µm) | Minimum local gap (µm) | Maximum local gap (µm) | *h_max_/h_min_* | Approx. local conductance ratio (*h_max_/h_min_*)^3^ |
| --- | --- | --- | --- | --- | --- | --- |
| L1 wide liner | 450.0 | 0 | 450.0 | 450.0 | 1.000 | 1.000 |
| L1 wide liner | 450.0 | 25 | 425.0 | 475.0 | 1.118 | 1.396 |
| L1 wide liner | 450.0 | 45 | 405.0 | 495.0 | 1.222 | 1.826 |
| L2 narrow liner | 50.00 | 0 | 50.00 | 50.00 | 1.000 | 1.000 |
| L2 narrow liner | 50.00 | 25 | 25.00 | 75.00 | 3.000 | 27.00 |
| L2 narrow liner | 50.00 | 45 | 5.000 | 95.00 | 19.00 | 6859 |

The sensitivity calculation shows that eccentricity is expected to be much more critical in the narrow liner. For the 1.2 mm liner, a 25 µm offset changes the local gap from 25 to 75 µm, corresponding to an approximate local conductance ratio of 27. A 45 µm offset gives a minimum gap of only 5 µm and an approximate local conductance ratio above 6800. These values are not a CFD prediction; they illustrate why local velocity differentials and mechanical alignment may become important in the narrow geometry.

Figure S4. Sensitivity of the approximate local conductance ratio to fiber eccentricity. The narrow L2 geometry is highly sensitive because the nominal radial clearance is only 50 µm.

## D) R-Script for the calculations

#!/usr/bin/env Rscript

\# ==============================================================================

\# Supplementary Note S1

\# Simplified numerical assessment of SPME Arrow liner geometry

\# ==============================================================================

\#

\# This R script performs all calculations used for the supplementary numerical

\# assessment of the SPME Arrow injection geometry and generates the corresponding

\# tables and plots.

\#

\# IMPORTANT:

\# This is NOT a validated CFD simulation. It is a simplified, comparative

\# geometrical and pneumatic calculation. It estimates:

\#

\#	1. Annular cross-sectional area

\#	2. Apparent linear carrier-gas velocity

\#	3. Residence time over the 20 mm coating length and 105 mm liner length

\#	4. Reynolds number

\#	5. Steady-state pressure drop for concentric laminar annular flow

\#	6. Eccentricity sensitivity using a narrow-gap approximation

\#

\# The pressure-drop calculation describes steady-state annular flow only.

\# It does NOT describe the transient pressure pulse during SPME Arrow insertion.

\#

\# The eccentricity calculation is only a sensitivity estimate. It is NOT a full

\# eccentric annular-flow solution.

\#

\# No external R packages are required.

\#

\# Run in R or RStudio:

\#

\#	source("SPME_Arrow_All_Supplementary_Calculations_with_Plots.R")

\#

\# Output directory:

\#

\#	./SPME_Arrow_Supplementary_R_Output

\#

\# ==============================================================================

\# ==============================================================================

\# 1. Input parameters

\# ==============================================================================

\# Temperatures

T_inlet_C	<- 23.0

T_injector_C <- 273.0

T_inlet_K	<- T_inlet_C + 273.15

T_injector_K <- T_injector_C + 273.15

\# Ideal-gas volumetric expansion factor from inlet gas temperature to injector

\# temperature under a constant-pressure approximation.

thermal_expansion_factor <- T_injector_K / T_inlet_K

\# Carrier gas and physical constants

carrier_gas <- "Helium"

P_Pa <- 101325.0	# pressure used for helium density estimate

R	<- 8.314462618	# universal gas constant, J mol^-1 K^-1

M_He <- 4.002602e-3	# molar mass of helium, kg mol^-1

\# Helium density at injector temperature and 1 atm from ideal gas law:

\# rho = P*M/(R*T)

rho_He_273_kg_m3 <- P_Pa * M_He / (R * T_injector_K)

\# Helium dynamic viscosity at injector temperature using Sutherland-type relation.

\# Reference values for helium:

mu_ref_Pa_s <- 1.864e-5	# dynamic viscosity at T_ref, Pa s

T_ref_K	<- 273.15

S_He_K	<- 79.4

mu_He_273_Pa_s <- mu_ref_Pa_s *

(T_injector_K / T_ref_K)^1.5 *

(T_ref_K + S_He_K) /

(T_injector_K + S_He_K)

\# Geometry

SPME_Arrow_OD_mm <- 1.10

liner_table <- data.frame(

Geometry	= c("L1 wide liner", "L2 narrow liner"),

Liner_ID_mm = c(2.00, 1.20),

stringsAsFactors = FALSE

)

coating_length_mm <- 20.0

liner_length_mm	<- 105

\# Nominal flow rates set at 23 deg C

flow_rates_mL_min <- c(0.8, 1.6, 2.4)

\# Eccentricities for sensitivity calculation

eccentricity_um <- c(0, 25, 45)

\# Output directory

output_dir <- "SPME_Arrow_Supplementary_R_Output"

dir.create(output_dir, showWarnings = FALSE, recursive = TRUE)

\# ==============================================================================

\# 2. Helper functions

\# ==============================================================================

annular_area_mm2 <- function(D_liner_mm, D_arrow_mm) {

pi / 4 * (D_liner_mm^2 - D_arrow_mm^2)

}

radial_clearance_mm <- function(D_liner_mm, D_arrow_mm) {

(D_liner_mm - D_arrow_mm) / 2

}

hydraulic_diameter_mm <- function(D_liner_mm, D_arrow_mm) {

\# For a concentric annulus:

\# Dh = D_outer - D_inner

D_liner_mm - D_arrow_mm

}

flow_mL_min_to_mm3_s <- function(flow_mL_min) {

\# 1 mL = 1000 mm^3

flow_mL_min * 1000 / 60

}

expanded_flow_mm3_s <- function(flow_mL_min, expansion_factor) {

flow_mL_min_to_mm3_s(flow_mL_min) * expansion_factor

}

apparent_velocity_mm_s <- function(Q_mm3_s, A_mm2) {

Q_mm3_s / A_mm2

}

residence_time_s <- function(length_mm, velocity_mm_s) {

length_mm / velocity_mm_s

}

reynolds_number <- function(rho_kg_m3, velocity_mm_s, Dh_mm, mu_Pa_s) {

velocity_m_s <- velocity_mm_s / 1000

Dh_m <- Dh_mm / 1000

rho_kg_m3 * velocity_m_s * Dh_m / mu_Pa_s

}

pressure_drop_annulus_Pa <- function(flow_mL_min, D_liner_mm, D_arrow_mm,

length_mm, mu_Pa_s, expansion_factor) {

\# Steady-state pressure drop for fully developed laminar flow in a concentric

\# annulus.

\#

\# Q = (pi * dp/dx / (8*mu)) *

\#	[Ro^4 - Ri^4 - ((Ro^2 - Ri^2)^2 / log(Ro/Ri))]

\#

\# Rearranged:

\# dp = Q * 8*mu*L / (pi * bracket)

\#

\# Q is corrected for thermal expansion from inlet gas temperature to injector

\# temperature under a constant-pressure approximation.

Q_m3_s <- flow_mL_min * 1e-6 / 60 * expansion_factor

Ro_m <- (D_liner_mm / 1000) / 2

Ri_m <- (D_arrow_mm / 1000) / 2

L_m <- length_mm / 1000

bracket <- Ro_m^4 - Ri_m^4 -

((Ro_m^2 - Ri_m^2)^2 / log(Ro_m / Ri_m))

dp_Pa <- Q_m3_s * 8 * mu_Pa_s * L_m / (pi * bracket)

dp_Pa

}

local_gap_parameters <- function(c_um, e_um) {

\# Narrow-gap eccentricity approximation:

\#

\#	h(theta) = c + e*cos(theta)

\#

\# Minimum and maximum gap:

\#

\#	h_min = c - e

\#	h_max = c + e

\#

\# Local hydraulic conductance is approximated to scale with h^3.

\#

\# This is only a sensitivity estimate.

h_min_um <- c_um - e_um

h_max_um <- c_um + e_um

if (h_min_um <= 0) {

ratio_h <- Inf

conductance_ratio <- Inf

} else {

ratio_h <- h_max_um / h_min_um

conductance_ratio <- ratio_h^3

}

data.frame(

Minimum_local_gap_um = h_min_um,

Maximum_local_gap_um = h_max_um,

hmax_hmin = ratio_h,

Conductance_ratio_h3 = conductance_ratio,

stringsAsFactors = FALSE

)

}

\# ==============================================================================

\# 3. Table S1: Input parameters

\# ==============================================================================

table_S1 <- data.frame(

Parameter = c(

"Carrier gas",

"SPME Arrow outer diameter used in model",

"Coating length",

"Wide liner L1 inner diameter",

"Narrow liner L2 inner diameter",

"Thermo Trace SSL liner length",

"Carrier-gas inlet temperature",

"Injector temperature",

"Thermal expansion factor Tinj/Tin",

"Helium density at 273 deg C and 1 atm",

"Helium dynamic viscosity estimated at 273 deg C"

),

Value = c(

carrier_gas,

paste0(SPME_Arrow_OD_mm, " mm"),

paste0(coating_length_mm, " mm"),

paste0(liner_table$Liner_ID_mm[liner_table$Geometry == "L1 wide liner"], " mm"),

paste0(liner_table$Liner_ID_mm[liner_table$Geometry == "L2 narrow liner"], " mm"),

paste0(liner_length_mm, " mm"),

paste0(T_inlet_C, " deg C"),

paste0(T_injector_C, " deg C"),

sprintf("%.3f", thermal_expansion_factor),

paste0(sprintf("%.4f", rho_He_273_kg_m3), " kg m^-3"),

paste0(sprintf("%.2e", mu_He_273_Pa_s), " Pa s")

),

stringsAsFactors = FALSE

)

\# ==============================================================================

\# 4. Table S2: Geometrical parameters

\# ==============================================================================

table_S2 <- data.frame()

for (i in seq_len(nrow(liner_table))) {

D_liner <- liner_table$Liner_ID_mm[i]

A <- annular_area_mm2(D_liner, SPME_Arrow_OD_mm)

gap <- radial_clearance_mm(D_liner, SPME_Arrow_OD_mm)

Dh <- hydraulic_diameter_mm(D_liner, SPME_Arrow_OD_mm)

row <- data.frame(

Geometry = liner_table$Geometry[i],

Liner_ID_mm = D_liner,

SPME_Arrow_OD_mm = SPME_Arrow_OD_mm,

Radial_clearance_mm = gap,

Annular_area_mm2 = A,

Hydraulic_diameter_mm = Dh,

stringsAsFactors = FALSE

)

table_S2 <- rbind(table_S2, row)

}

area_L2 <- table_S2$Annular_area_mm2[table_S2$Geometry == "L2 narrow liner"]

table_S2$Area_ratio_vs_L2 <- table_S2$Annular_area_mm2 / area_L2

\# ==============================================================================

\# 5. Table S3: Apparent velocity and residence time

\# ==============================================================================

table_S3 <- data.frame()

for (i in seq_len(nrow(liner_table))) {

D_liner <- liner_table$Liner_ID_mm[i]

A <- annular_area_mm2(D_liner, SPME_Arrow_OD_mm)

for (flow in flow_rates_mL_min) {

Q_23 <- flow_mL_min_to_mm3_s(flow)

Q_273 <- expanded_flow_mm3_s(flow, thermal_expansion_factor)

u_23 <- apparent_velocity_mm_s(Q_23, A)

u_273 <- apparent_velocity_mm_s(Q_273, A)

row <- data.frame(

Geometry = liner_table$Geometry[i],

Flow_mL_min = flow,

Q_at_23C_mm3_s = Q_23,

Q_expanded_to_273C_mm3_s = Q_273,

u_app_at_23C_mm_s = u_23,

u_app_at_273C_mm_s = u_273,

Residence_time_over_20mm_s = residence_time_s(coating_length_mm, u_273),

Residence_time_over_78p5mm_s = residence_time_s(liner_length_mm, u_273),

stringsAsFactors = FALSE

)

table_S3 <- rbind(table_S3, row)

}

}

\# ==============================================================================

\# 6. Table S4: Reynolds numbers and pressure-drop estimates

\# ==============================================================================

table_S4 <- data.frame()

for (i in seq_len(nrow(liner_table))) {

D_liner <- liner_table$Liner_ID_mm[i]

A <- annular_area_mm2(D_liner, SPME_Arrow_OD_mm)

Dh <- hydraulic_diameter_mm(D_liner, SPME_Arrow_OD_mm)

for (flow in flow_rates_mL_min) {

Q_273 <- expanded_flow_mm3_s(flow, thermal_expansion_factor)

u_273 <- apparent_velocity_mm_s(Q_273, A)

Re <- reynolds_number(

rho_kg_m3 = rho_He_273_kg_m3,

velocity_mm_s = u_273,

Dh_mm = Dh,

mu_Pa_s = mu_He_273_Pa_s

)

dP_20 <- pressure_drop_annulus_Pa(

flow_mL_min = flow,

D_liner_mm = D_liner,

D_arrow_mm = SPME_Arrow_OD_mm,

length_mm = coating_length_mm,

mu_Pa_s = mu_He_273_Pa_s,

expansion_factor = thermal_expansion_factor

)

dP_785 <- pressure_drop_annulus_Pa(

flow_mL_min = flow,

D_liner_mm = D_liner,

D_arrow_mm = SPME_Arrow_OD_mm,

length_mm = liner_length_mm,

mu_Pa_s = mu_He_273_Pa_s,

expansion_factor = thermal_expansion_factor

)

row <- data.frame(

Geometry = liner_table$Geometry[i],

Flow_mL_min = flow,

Reynolds_number_at_273C = Re,

Estimated_dP_over_20mm_Pa = dP_20,

Estimated_dP_over_78p5mm_Pa = dP_785,

Estimated_dP_over_78p5mm_mbar = dP_785 / 100,

stringsAsFactors = FALSE

)

table_S4 <- rbind(table_S4, row)

}

}

\# ==============================================================================

\# 7. Table S5: Eccentricity sensitivity

\# ==============================================================================

table_S5 <- data.frame()

for (i in seq_len(nrow(liner_table))) {

D_liner <- liner_table$Liner_ID_mm[i]

c_um <- radial_clearance_mm(D_liner, SPME_Arrow_OD_mm) * 1000

for (e_um in eccentricity_um) {

\# Only physically meaningful if e < c

if (e_um >= c_um) {

gap_par <- data.frame(

Minimum_local_gap_um = c_um - e_um,

Maximum_local_gap_um = c_um + e_um,

hmax_hmin = Inf,

Conductance_ratio_h3 = Inf,

stringsAsFactors = FALSE

)

} else {

gap_par <- local_gap_parameters(c_um, e_um)

}

row <- data.frame(

Geometry = liner_table$Geometry[i],

Nominal_radial_clearance_um = c_um,

Eccentricity_e_um = e_um,

Minimum_local_gap_um = gap_par$Minimum_local_gap_um,

Maximum_local_gap_um = gap_par$Maximum_local_gap_um,

hmax_hmin = gap_par$hmax_hmin,

Approx_local_conductance_ratio_hmax_hmin_cubed = gap_par$Conductance_ratio_h3,

stringsAsFactors = FALSE

)

table_S5 <- rbind(table_S5, row)

}

}

\# ==============================================================================

\# 8. Save all tables

\# ==============================================================================

write.csv(table_S1, file.path(output_dir, "Table_S1_input_parameters.csv"), row.names = FALSE)

write.csv(table_S2, file.path(output_dir, "Table_S2_geometry.csv"), row.names = FALSE)

write.csv(table_S3, file.path(output_dir, "Table_S3_velocity_residence_time.csv"), row.names = FALSE)

write.csv(table_S4, file.path(output_dir, "Table_S4_reynolds_pressure_drop.csv"), row.names = FALSE)

write.csv(table_S5, file.path(output_dir, "Table_S5_eccentricity_sensitivity.csv"), row.names = FALSE)

\# ==============================================================================

\# 9. Plotting functions

\# ==============================================================================

\# These plotting functions use Base R only.

\# They save publication-style PNG files and, in an interactive R session,

\# also display the plots in the active graphics device.

plot_velocity <- function() {

y <- table_S3$u_app_at_273C_mm_s

plot(

NA,

xlim = range(flow_rates_mL_min),

ylim = range(y) * c(0.95, 1.05),

xlab = expression("Carrier-gas flow (mL min"^-1*")"),

ylab = expression("Apparent linear velocity at 273 "*degree*"C (mm s"^-1*")"),

main = "Calculated apparent linear carrier-gas velocity"

)

pch_values <- c(16, 17)

lty_values <- c(1, 1)

for (i in seq_len(nrow(liner_table))) {

geom <- liner_table$Geometry[i]

dat <- table_S3[table_S3$Geometry == geom, ]

lines(

dat$Flow_mL_min,

dat$u_app_at_273C_mm_s,

type = "o",

pch = pch_values[i],

lty = lty_values[i]

)

}

legend(

"topleft",

legend = liner_table$Geometry,

pch = pch_values,

lty = lty_values,

bty = "n"

)

}

plot_residence_time <- function() {

y <- table_S3$Residence_time_over_20mm_s

plot(

NA,

xlim = range(flow_rates_mL_min),

ylim = range(y),

log = "y",

xlab = expression("Carrier-gas flow (mL min"^-1*")"),

ylab = "Estimated residence time over 20 mm (s)",

main = "Estimated residence time over 20 mm"

)

pch_values <- c(16, 17)

lty_values <- c(1, 1)

for (i in seq_len(nrow(liner_table))) {

geom <- liner_table$Geometry[i]

dat <- table_S3[table_S3$Geometry == geom, ]

lines(

dat$Flow_mL_min,

dat$Residence_time_over_20mm_s,

type = "o",

pch = pch_values[i],

lty = lty_values[i]

)

}

legend(

"topright",

legend = liner_table$Geometry,

pch = pch_values,

lty = lty_values,

bty = "n"

)

}

plot_pressure_drop <- function() {

y <- table_S4$Estimated_dP_over_78p5mm_Pa

plot(

NA,

xlim = range(flow_rates_mL_min),

ylim = range(y),

log = "y",

xlab = expression("Carrier-gas flow (mL min"^-1*")"),

ylab = "Estimated steady-state pressure drop over 105 mm (Pa)",

main = "Approximate steady-state annular pressure drop"

)

pch_values <- c(16, 17)

lty_values <- c(1, 1)

for (i in seq_len(nrow(liner_table))) {

geom <- liner_table$Geometry[i]

dat <- table_S4[table_S4$Geometry == geom, ]

lines(

dat$Flow_mL_min,

dat$Estimated_dP_over_78p5mm_Pa,

type = "o",

pch = pch_values[i],

lty = lty_values[i]

)

}

legend(

"topleft",

legend = liner_table$Geometry,

pch = pch_values,

lty = lty_values,

bty = "n"

)

}

plot_eccentricity <- function() {

y <- table_S5$Approx_local_conductance_ratio_hmax_hmin_cubed

plot(

NA,

xlim = range(eccentricity_um),

ylim = range(y[is.finite(y)]),

log = "y",

xlab = expression("Fiber eccentricity e ("*mu*"m)"),

ylab = expression("Approx. local conductance ratio ("*h[max]/h[min]*")"^3),

main = "Sensitivity to fiber eccentricity"

)

pch_values <- c(16, 17)

lty_values <- c(1, 1)

for (i in seq_len(nrow(liner_table))) {

geom <- liner_table$Geometry[i]

dat <- table_S5[table_S5$Geometry == geom, ]

lines(

dat$Eccentricity_e_um,

dat$Approx_local_conductance_ratio_hmax_hmin_cubed,

type = "o",

pch = pch_values[i],

lty = lty_values[i]

)

}

legend(

"topleft",

legend = liner_table$Geometry,

pch = pch_values,

lty = lty_values,

bty = "n"

)

}

\# ==============================================================================

\# 10. Save plots as PNG

\# ==============================================================================

png(file.path(output_dir, "Figure_S1_velocity_vs_flow.png"),

width = 1800, height = 1200, res = 300)

plot_velocity()

dev.off()

png(file.path(output_dir, "Figure_S2_residence_time_20mm.png"),

width = 1800, height = 1200, res = 300)

plot_residence_time()

dev.off()

png(file.path(output_dir, "Figure_S3_pressure_drop.png"),

width = 1800, height = 1200, res = 300)

plot_pressure_drop()

dev.off()

png(file.path(output_dir, "Figure_S4_eccentricity.png"),

width = 1800, height = 1200, res = 300)

plot_eccentricity()

dev.off()

\# ==============================================================================

\# 11. Plot interactively when run in RStudio / interactive R

\# ==============================================================================

if (interactive()) {

cat("\nInteractive R session detected. Plotting figures to the active graphics device.\n\n")

plot_velocity()

readline(prompt = "Press <Enter> for Figure S2...")

plot_residence_time()

readline(prompt = "Press <Enter> for Figure S3...")

plot_pressure_drop()

readline(prompt = "Press <Enter> for Figure S4...")

plot_eccentricity()

}

\# ==============================================================================

\# 12. Console summary

\# ==============================================================================

cat("\nSupplementary numerical assessment completed.\n")

cat("Output directory:", normalizePath(output_dir), "\n\n")

cat("Thermal expansion factor Tinj/Tin:", thermal_expansion_factor, "\n")

cat("Helium density at 273 deg C and 1 atm:", rho_He_273_kg_m3, "kg/m^3\n")

cat("Helium dynamic viscosity at 273 deg C:", mu_He_273_Pa_s, "Pa s\n\n")

cat("Table S1: Input parameters\n")

print(table_S1)

cat("\nTable S2: Geometry\n")

print(table_S2)

cat("\nTable S3: Apparent velocity and residence time\n")

print(table_S3)

cat("\nTable S4: Reynolds numbers and pressure-drop estimates\n")

print(table_S4)

cat("\nTable S5: Eccentricity sensitivity\n")

print(table_S5)

cat("\nFiles written:\n")

print(list.files(output_dir, full.names = TRUE))

cat("\nNote:\n")

cat("The pressure-drop calculation is a steady-state concentric annular-flow estimate.\n")

cat("It does not describe the transient pressure pulse during SPME Arrow insertion.\n")

cat("The eccentricity calculation is a narrow-gap sensitivity approximation only.\n")
